# *“Nothing is going to replace an in-person visit”*: Canadian long-term care providers’ and recipients’ perspectives on when telehealth for physician visits is not appropriate

**DOI:** 10.1186/s44247-025-00219-8

**Published:** 2025-10-27

**Authors:** Tyler R. Cole, Valorie A. Crooks, Janice Sorensen, Sherin Jamal, Akber Mithani, Lillian Hung, Jeremy Snyder, Catherine Youngren

**Affiliations:** 1 Department of Geography, Simon Fraser University, Burnaby, BC Canada; 2 Long-Term Care and Assisted Living, Fraser Health Authority, Surrey, BC Canada; 3 School of Nursing, University of British Columbia, Vancouver, BC Canada; 4 Faculty of Health Sciences, Simon Fraser University, Burnaby, BC Canada; 5 Fraser Health Long-Term Care and Assisted Living Research Partners Group, Surrey, BC Canada

**Keywords:** Long-term care, Telehealth, Appropriateness, Residents, Physicians, Caregivers, Staff

## Abstract

**Background:**

Within long-term care (LTC) homes, telehealth use has been found to reduce unnecessary emergency department transfers, support the care needs of rural and underserved communities, and supplement in-person physician care. Despite these benefits, it is not well understood when telehealth is not an appropriate medium for providing physician care to residents with complex health needs. This knowledge gap must be addressed given the recent rise in telehealth use in LTC homes in many health systems following the COVID-19 pandemic, when virtual care use increased in many health care sectors to limit travel and in-person exposure risks, that is expected to be maintained going forward.

**Methods:**

This analysis contributes to a broader evaluative study investigating care provider and care recipient experiences and preferences for physician telehealth in LTC homes within the Fraser Health region in British Columbia, Canada. For data collection, semi-structured interviews and focus groups were undertaken with seventy care providers (staff, physicians) and recipients (residents, family caregivers). Using a thematic approach, transcripts were analyzed to find common instances when using telehealth for physician care was seen as not appropriate across participant groups.

**Results:**

Three types of patient care activities were identified as not appropriate to be conducted via physician visits using telehealth. First, new patient visits were thought to benefit from an interpersonal and conversational familiarity that could not be supported by telehealth. Second, difficult in-depth conversations that required conversational nuance (e.g., eye contact, supportive body language), such as palliative care planning, were thought to be inappropriate for telehealth appointments. Finally, instances where LTC staff would need to perform hands-on clinical assessments on behalf of physicians who were attending virtually via telehealth were not seen as desirable.

**Conclusions:**

This analysis highlights perspectives surrounding when telehealth is not appropriate for providing physician services for residents in LTC based on the preferences and experiences shared by both care recipients and care providers. The findings present an opportunity to develop and implement guidelines on appropriate use of telehealth in LTC to support best care practices.

## Background

Long-term care (LTC) homes are residential care settings where staff provide continuing care to support the personal and medical needs of residents who are unable to live independently [1, 2]. LTC residents typically have high rates of cognitive impairment, frailty, and palliative care needs [3, 4]. While LTC homes typically do not have age restrictions, residents in the Canadian province of British Columbia (BC), which is the focus of the current analysis, are on average 83 years of age [4]. To meet residents’ individualized care needs, LTC homes in BC employ a variety of on-site care providers such as, but not limited to, care aides, nurses, physical, occupational, and recreational therapists, registered dieticians, and social workers [5, 6]. Further, each home has an associated attending physician who oversees the medical care of all residents and is available to visit with residents as needed to support their health and wellbeing [7]. While care services and associated support staff may differ between LTC homes across BC, those that are publicly funded and affiliated with regional health authorities generally follow a similar staffing composition.

Telehealth involves using virtual methods, most commonly video and phone calls, to remotely monitor and provide care for patients [8]. In LTC settings, telehealth care is often administered within a residents’ room, however, in the case of infrastructure difficulties such as poor Wi-Fi connectivity it may occur in another suitable space within the home [9]. Until recently, telehealth in LTC homes was most commonly used to avoid unnecessary emergency department transfers by enabling physicians to call in virtually to make clinical assessments [10–12]. For example, this technology reduced the chance of potential infections for residents associated with being hospitalized [13]. LTC homes in rural and remote areas have also benefited from telehealth as this care medium has improved access to physician and specialist care for residents [14]. During the COVID-19 pandemic, LTC homes throughout BC and across Canada enacted strict visitation restrictions to safeguard residents against the virus [15, 16]. Consequently, there was a significant increase in telehealth use to ensure that residents’ health needs were being met despite the minimized on-site presence of physicians [14, 17]. Physicians across many care sectors in Canada who shifted to using telehealth as a primary form of care delivery during this period have indicated an intention to continue using it at rates greater than they did prior to the pandemic [18, 19]. This is true in BC’s LTC sector, where telehealth is no longer thought of as a secondary or supplementary medium for care delivery by physicians.

Research has documented some instances in which telehealth use is both common and desired in the LTC context, most of which are intended to supplement in-person physician care [20]. For example, one common use is for physicians to regularly review medications being taken by residents via phone or video [21]. Some physical assessments are successfully conducted by telehealth, such as monitoring wound care [22], assessing spasticity [23], and orthopedic consultations [24]. For mental health assessments, telehealth has been used for diagnosing and evaluating dementia-related psychosis [25] and other psychiatric consultations to improve quality of life [26]. Great uncertainties remain, however, regarding instances in which it is not appropriate or desired to use telehealth for providing physician care for LTC residents and their complex medical and social support needs. This draws particular concern given the pressing need to support best practice in light of the sustained, more-than-supplementary use of telehealth by physicians in the LTC sector post-pandemic.

The qualitative analysis presented herein responds directly to the knowledge gap identified above by integrating the perspectives of LTC care providers and recipients alike to explore specific instances when telehealth for physician visits is understood to be undesirable or inappropriate. We draw together insights from staff, physicians, residents, and family caregivers involved in the LTC sector in the populous Fraser Health Authority administrative region of BC. While some existing literature has looked at telehealth appropriateness in the LTC context e.g [27–29], it is still quite limited and perspectives from care recipients are severely underrepresented. The current analysis not only gives voice to this underrepresented group, but also considers appropriateness at a very granular level by acknowledging different types or purposes of physician visits that take place in LTC. As such, the findings will be used by decision-makers within the Fraser Health Authority and other knowledge users across BC to inform best practice planning and implementation regarding when telehealth is not appropriate and in-person physician care is best to support LTC residents’ complex care needs.

## Methods

The current analysis is part of a larger qualitative evaluation study that has retrospectively investigated the rapid roll-out of telehealth services to support physician visits implemented in LTC homes at the outset of the COVID-19 pandemic. The evaluation has also explored interest in maintaining or increasing telehealth use in LTC homes in the region post-pandemic, identifying lessons from the rapid roll-out period for continued use. The area of focus of the evaluation is the Fraser Health Authority region of BC, which houses 83 LTC homes. The study was designed using Patton’s (2008) 12-step utilization-focused evaluation process [30]. The evaluative steps required us to: consider the readiness for the evaluative focus; consider the readiness of the evaluative team; engage end users; conduct a situational analysis; identify participant groups; define the evaluative scope; choose data collection techniques; pilot data collection techniques; collect data; analyze data; mobilize knowledge; and critically reflect [30]. Utilization-focused evaluation requires an integrated knowledge translation approach as it emphasizes the collaborative efforts between researchers and end-users to ensure the relevance of findings for future decision-making [30]. The Fraser Health Long-Term Care and Assisted Living Research Partners Group, which includes LTC residents, family members, volunteers, and staff, was involved throughout the evaluation to ensure an engaged and patient-centered approach was integrated into every component. The conceptual framing of the evaluation was informed by Canada Health Infoway’s Benefits Evaluation – Clinical Adoption framework and Canada’s Quadruple Aim for Strengthening healthcare systems [31, 32]. The former identifies micro- and meso-level factors that contribute to high-quality virtual care, including telehealth, while the latter identifies enhancing patient experiences, health outcomes, care costs, and healthcare work environments as priorities for creating a strengthened healthcare system.

Four participant groups involved in care provision or receipt in Fraser Health’s LTC sector were consulted in this study: residents, family caregivers, providers, and physicians. *Residents* lived in LTC homes and could participate in one-on-one interviews or dyadic interviews with a family caregiver. *Family caregivers* were friends and/or family of those living in LTC homes who took on some informal care responsibilities and could participate in one-on-one or dyadic interviews. *Care staff* worked in LTC homes providing direct care, such as Care Aides and Licensed Practical Nurses, or administrators, such as Directors of Care, and could participate in one-on-one interviews. *Physicians* provided medical care to LTC residents, including via telehealth, and could participate in a one-on-one interview or a virtual focus group. The virtual focus group option was only held for physicians to accommodate their time-constraints and to provide a neutral interview site. We aimed to recruit at least 70 participants across these groups, seeking to hear from at least 30 care recipients (residents and family caregivers) and 40 care providers (care staff and physicians) from a number of LTC homes across the Fraser Health Authority region. Data was collected over a 6-month period (March to August, 2023).

To recruit care recipients (namely residents and family caregivers) and care staff, e-mails inviting participation were sent to Directors of Care and Medical Directors in each of the 83 LTC homes in the Fraser Health Authority region. The emails contained information about the study and an invitation for staff, residents and caregivers to participate. Posters were also provided that could be displayed in common areas of the LTC home. All recruitment materials were provided in English with the option to request materials in other languages commonly used in the region – namely French, Korean, Punjabi, Farsi, Spanish, Chinese, and Vietnamese. To support physician participation, an invitation was sent to LTC physicians to participate in a virtual focus group immediately following a regional physician leadership team meeting. The study was further advertised to care recipients and care staff on REACH BC, a website where volunteers can sign up to participate in health research in BC.

To be eligible for the study, all participants needed to be cognitively able to participate in an interview and provide verbal consent. Residents and caregivers were not required to have firsthand experience with telehealth services but were recruited from homes where it was known that telehealth was being used for physician care. For care staff and physicians, it was expected they had experience in either supporting or providing telehealth services for physician visits in the LTC homes affiliated with the Fraser Health Authority. Those who met these inclusion criteria took part in a virtual (online or phone) or in-person interview or focus group and received an honorarium. All interviews and focus groups started with a series of demographic questions followed by the use of Nelson and colleagues’ (2022) validated Digital Health Care Literacy Scale [33]. This Scale is the sum of five-point Likert scale responses to 3-items assessing one’s ability to utilize different forms of technology and solve basic technological issues on their own [33]. The Scale was used verbatim as openly and originally published by the developers. The main part of the interview included questions about participants’ experiences of either receiving or providing telehealth for physician visits. Further, questions probed into participants’ preferences regarding physician telehealth, any barriers or enablers for this care medium, and their telehealth preferences going forward. The full interview guides are published in an article reporting on a separate analysis drawn from this same evaluative study [9]. Each interview was conducted either by the first author or a research assistant trained by the first author. The physician focus group was conducted by a senior member of the research team, with the first author serving as a notetaker. On average, each interview lasted 10 to 30 min while the focus group lasted approximately an hour. Interviews and focus groups were audio recorded and professionally transcribed verbatim.

Transcripts were reviewed by the lead author to remove personal identifiers and ensure completeness. Anonymized transcripts were uploaded into NVivo to manage data organization and coding. A thematic approach to analysis was employed, which was informed by Braun and Clark’s (2012) approach [34]. One member of the Fraser Health Long-Term Care and Assisted Living Research Partners Group and the research team each independently read three transcripts to provide initial input on themes and potential analytical directions to the research team. Three meta-themes were identified for deeper analysis. To further enhance rigour and to engage in best practice around end-integrated knowledge translation, the analytic scope of these meta-themes was presented back to the Long-Term Care and Assisted Living Research Partners Group to ensure that these directions were relevant to current end-users.

Themes and sub-themes associated with the three meta-themes were identified through an iterative process of transcript review and team discussion led by the lead author. The scope of each was confirmed by the second and third authors, after which a coding tree was created that integrated inductive and deductive codes. The dataset was coded by the first author to ensure interpretive consistency. Input from the second and third authors was sought to address any concerns that emerged during the coding process. Coding extracts were independently reviewed by members of the research team as a final step to confirm the integrity of the coding tree and its interpretation. The research team then met to discuss the details of the analytic directions for each of the meta-themes, two of which were identified to be most robust and ready to move to full analysis. One of these robust meta-themes serves as the focus of the current paper, while the other will be separately explored. Our next step was to contrast the themes central to this analysis against the existing literature to identify the novelty of the analytic directions and opportunities for transferability. This is an important step in thematic analysis [34]. We engage with such literature in the discussion section, while in the section that follows, we present the analytic findings. To support the trustworthiness of interpretation we integrate verbatim quotations throughout from both care provider and care recipient participant groups.

## Results

For this study, 70 participants were recruited from 27 LTC homes in the Fraser Health Authority region across the four groups: residents (*n* = 26), family caregivers (*n* = 13), care staff (*n* = 16), and physicians (*n* = 15). All participants completed one-on-one interviews expect for 8 physicians who were included in a single virtual focus group. On average, residents were 76 years of age (range: 47–90) and had lived in LTC for 3 years. Family caregivers were on average 52 years of age (range: 24–74). Care staff had worked in the LTC sector on average for 11 years while physicians had done so for 12 years. Using Statistics Canada’s ethnicity designations [35] the majority of participants identified as White (69%) while there were also smaller numbers who identified as Southeast Asian (16%), South Asian (7%), Indigenous (3%), Latin American (1%), and Middle Eastern (1%). Two participants chose not to disclose their ethnicities. Forty-two participants identified as women, 27 as men, and one as non-binary. A majority of residents and family caregivers reported having access to a personal device that they could use for telehealth for physician visits. However, residents were generally more apprehensive than family caregivers about using technology, and only four reported direct experience of having had physician visits via telehealth. Physicians and staff provided and supported care, respectively, using various methods of telehealth such as video and phone calls, including for physician visits. Physicians and staff felt comfortable using technology with the small exception that care staff indicated they were not as adept at solving basic technological problems as were physicians. Information about the digital literacy of participants is summarized in Tables 1 and 2, which provides important context for some of the preferences shared from care provider and care recipient groups in this section.

**Table 1:**
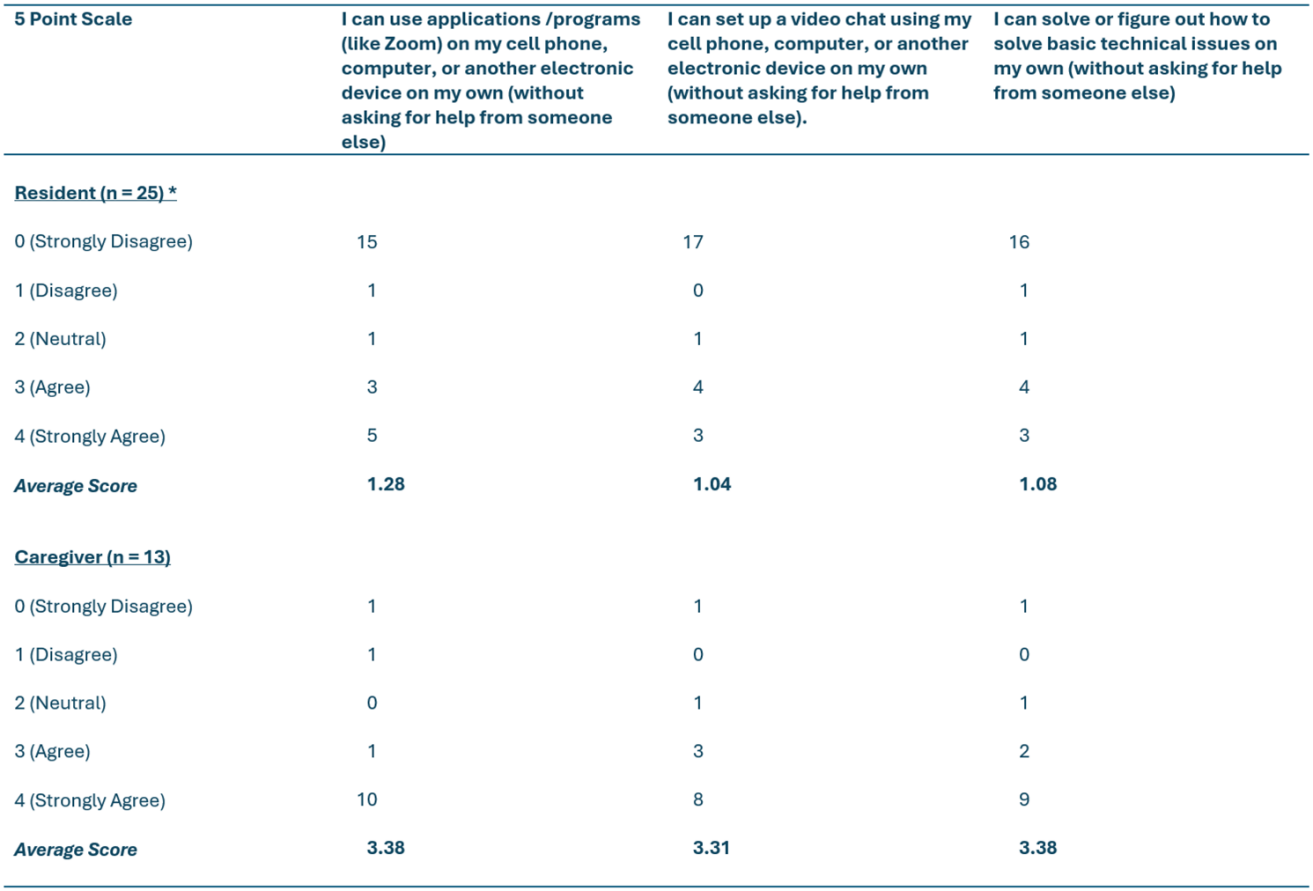
Digital literacy scores of care recipients based on Nelson and colleagues, 2022 digital health literacy scale (DHLS) [33]

Note: *One resident passed away during the study before we were able to revisit and do their digital literacy scores. As such, *n* = 25 participants

**Table 2:**
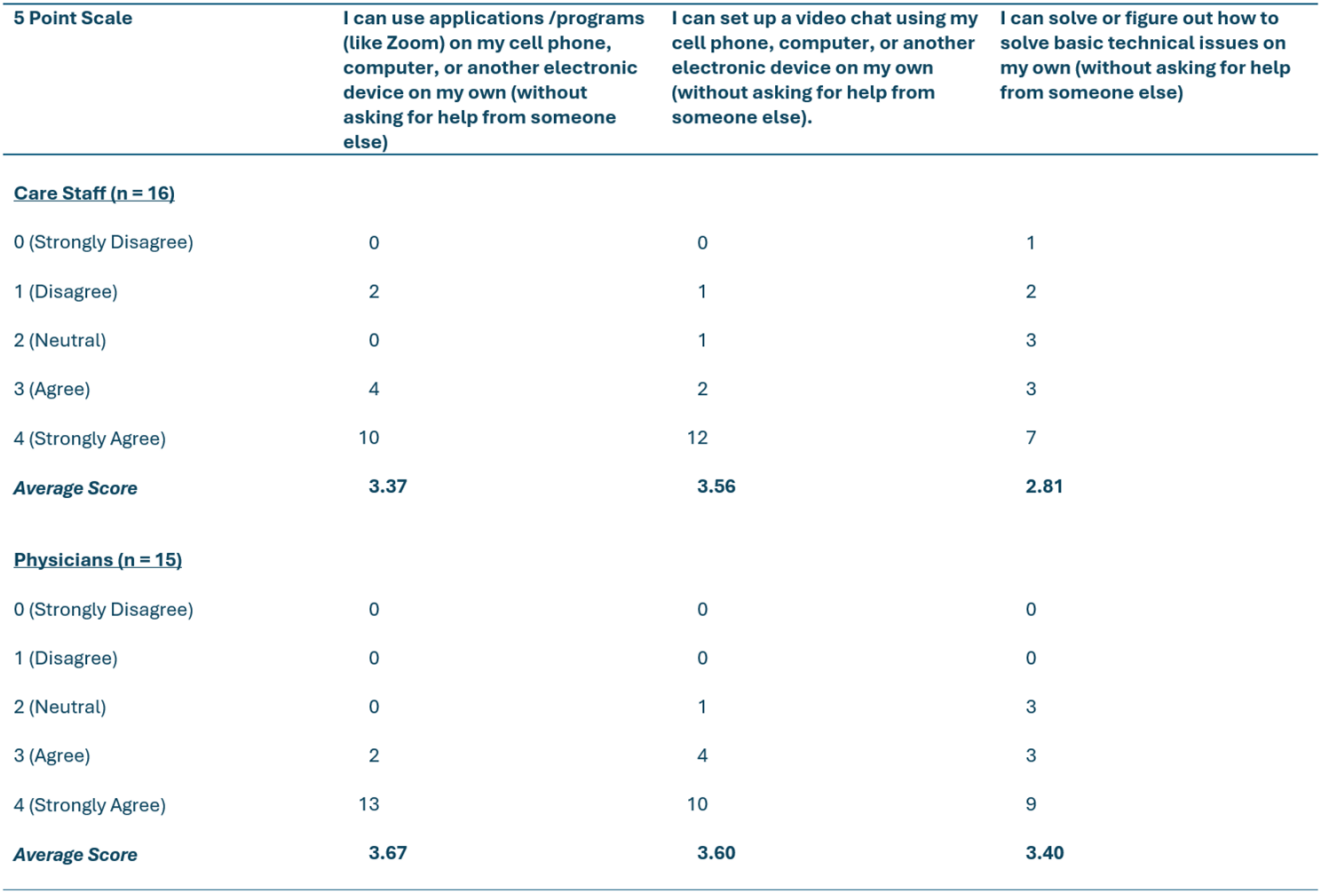
Digital literacy scores of care providers based on Nelson and colleagues, 2022 digital health literacy scale (DHLS) [33]

Participants across all four groups openly discussed their experiences, observations, and expectations of instances when physician telehealth was perceived as not appropriate. Through thematic analysis we identified three such instances that were discussed with consistency across participant groups: when new resident-physician introductions were being made; when in-depth and difficult conversations needed to be had; and when hands-on clinical assessments were required. Perspectives from care recipient groups and care provider groups offered unique experiential insights on why such instances were deemed not suitable for physician visits via telehealth in the LTC context. In the remainder of this section, we expand on each of these instances, contrasting perspectives from care recipient and provider groups to understand the full scope of why participants identified each as not being a suitable focus for telehealth appointments with physicians. Though we discuss them separately in the following sub-sections, we acknowledge that there are intersections between these instances, some of which we explore in the discussion section that follows.

### Care-recipient-physician introductions

Participants in all groups discussed the value of having in-person interactions between physicians and recipients when meeting for the first time. Residents who were admitted to LTC during the pandemic when in-person physician visits were limited, sometimes reported not knowing who their physician was despite widespread use of physician telehealth in their LTC home. It was thought that one reason for such confusion was that introductory meetings had not happened in person. For all participant groups, there was apprehension that not being able to have in-person new patient visits negatively impacted the development of interpersonal continuity of care.

For care recipients, the lack of an in-person introduction negatively impacted the development of a sense of personal connection and rapport with physicians. For some family caregivers, it was mentioned that not meeting directly with the physician providing care raised questions about how care decisions were being made. In some instances, family caregivers reported only ever meeting with physicians virtually in group contexts: *“out of all of the care conferences*,* the physician only participated in one or two. It would be great if they could be there more to ask more pointed questions or to have a dedicated phone call.”* It was explained that visiting with physicians only in group telehealth conferences was not an adequate substitute for the rapport built through an in-person introductory meeting, nor was communication being channeled exclusively by direct care staff to residents and family caregivers. Two of the four residents who had received physician care in LTC via telehealth mentioned that they felt they could not adequately express their concerns nor speak freely. The sentiment *“It’s just a blank conversation back and forth”* was shared by one resident referring to how difficult it was to explain their care needs over the phone to the physician.

The views of care providers were predominately aligned with care recipients with regard to the importance and value of having an initial in-person introductory meeting. Many care staff agreed that an initial in-person physician visit was important for residents since many struggled to actively participate in telehealth meetings due to hearing, visual, and/or cognitive impairments. Care staff also agreed that in-person introductory visits helped residents to get to know the physician responsible for their care. The importance of creating such an understanding was highlighted by care providers. For example, it was noted that it could be confusing for new residents to comprehend that a LTC home physician was now responsible for overseeing their medical care as opposed to the family physician they may have been visiting with in a community clinic for years. Among physicians, it was agreed that it was difficult to get to know residents and their families when having to start building a relationship using telehealth. As one physician put it *“you ultimately need the in-person interaction to establish that trust and the therapeutic relationship*,*”* while another explained that *“you have to form a rapport with someone before you can carry on with virtual visits.”* Comments such as these underscored the significance of shifting to telehealth only after first meeting in person.

### Difficult in-depth conversations

Participants across all groups agreed that difficult in-depth conversations, such as end-of-life care planning, were not appropriate to be done over telehealth. Participants placed a high value on the tone and dynamics of in-person interpersonal connections supporting the success of what could be very challenging and nuanced discussions involving several parties. Specifically, care providers and recipients alike noted that telehealth meetings with physicians and others in the care team did not allow for enough eye contact or personal touch to enable positive interactions during difficult in-depth conversations.

For care recipients, telehealth was generally viewed as useful for physicians to quickly consult with residents and not for in-depth extensive consultation and information exchange. For a majority of family caregivers, there was a preference for in-person discussions with physicians for difficult conversations such as end-of-life care planning or other emotionally laden conversations. In such instances, telehealth excluded the integration of caring and comforting body language. As one family caregiver put it: *“with those more serious conversations*,* I would prefer to have a closer type of dialogue instead of just a few minutes over the phone.”* Others related to this experience, adding that it was harder to ‘read the room’ when difficult conversations took place during telehealth visits. Some were uncomfortable discussing certain topics, such as end-of-life planning, in great depth through telehealth appointments with their physicians, signalling there was a lack of face-to-face contact and personal touch to support them. One resident added that *“because I’m not comfortable with technology*,*”* participating in any telehealth meetings, whether brief or in-depth, was not an option. This was not surprising given the difficulties residents reported regarding using the types of digital technologies and applications that can support telehealth in Table 1, which were much greater than those reported by staff and physicians in Table 2. A small number of residents were indifferent as to whether in-depth conversations happened in person or virtually, while some caregivers noted that in-person meetings augmented by telehealth could support the participation of remote family members.

Care providers echoed care recipients’ desires for in-person interactions for difficult in-depth conversations, noting the inappropriateness of telehealth for longer meetings with residents and caregivers. As a care staff explained: *“someone who’s going to palliative care or going to end-of-life*,* I really think that still having a physician being there and talking to family is helpful.”* Other care staff agreed, adding that having a physician physically present during such discussions reinforced personal connections between care providers and recipients. Most physicians indicated that they were content using telehealth for ongoing minor consults, such as medication reconciliation, but urged that having an in-person presence was still preferred for in-depth conversations. For example, one physician mentioned *“I personally find having those difficult conversations [with families] face-to-face much easier.”* Overall, it was agreed that there needed to be an appropriate balance of telehealth and in-person meetings depending on the nature of the physician visit.

### Advanced clinical assessments

Participants raised concerns about the feasibility and quality of performing hands-on clinical assessments through telehealth appointments through physicians instructing nursing staff to assess on their behalf. While specific concerns varied by participant group, both care recipients and caregivers questioned the appropriateness of telehealth for clinical assessment. There was consensus that routine assessments, such as blood pressure checks, were suitable for physician telehealth appointments. Alternatively, assessments that required more advanced clinical skills, and thereby necessitated extensive physician guidance for care staff not trained to independently conduct the assessment, were best to be done in person to ensure quality.

Many residents did not note specific preferences for how their physical assessments were done. Instead, they were primarily concerned about how conversations unfolded when care was delivered via telehealth, including during physical assessments. Family caregivers, however, were more vocal about the inappropriateness of using telehealth for physical assessments and diagnostic testing. In a few cases, family caregivers were okay with having minor assessments done via telehealth appointments, such as examining rashes or small wounds using video call functions. Overall, the majority felt that physicians should be present and not reliant on care staff to conduct advanced clinical assessments. The statement, *“as far as I’m concerned*,* with a vulnerable population*,* nothing is going to replace an in-person visit*,*”* aligned with what was said by many caregivers. This was further stressed by another caregiver who explained that *“I didn’t really like the virtual or phone because you can’t diagnose someone by not seeing them and some of the changes may not always be describable.”* Many caregivers were specifically concerned that new symptoms would go unnoticed or undiagnosed if a physician was not present during a clinical assessment.

Care staff mainly echoed the concerns raised by family caregivers, noting that telehealth was inappropriate for physician visits that required hands-on clinical assessment. One main concern for care staff was being regularly tasked with the responsibility for verbally or visually relaying all details about residents’ symptoms or being guided by physicians to undertake advanced clinical assessments, which many cited as being challenging. As one care staff participant explained:

*…in terms of consults with a doctor*,* it’s so hard. I mean you can describe it in your own way*,* but even if you’re doing a video call with them it’s hard to distinguish the width and size of a laceration for example. It’s so different between in-person versus a picture or video*

While there were a few physicians who were comfortable with guiding nurses through virtual clinical assessments, most preferred to conduct such consults in person. They specifically felt that telehealth was not an appropriate medium for being able to fully examine a resident and provide an accurate diagnosis in cases requiring more advanced clinical assessment. As one physician put it, *“We were relying on nurses who do not have the same scope of practice. They play an important role*,* but we were expecting them to be physician assistants [in instances of telehealth appointments].”* Outside of crisis circumstances, such as during limited in-person contact during the COVID-19 pandemic or home closures due to environmental disasters (e.g., nearby flooding), they agreed that telehealth involving advanced clinical assessments were not a suitable replacement for in-person visits.

## Discussion

Our findings have highlighted resident, family caregiver, staff and physician groups’ experiences and expectations with telehealth in LTC and their preferences and perspectives on the appropriateness of this care medium for physician visits going forward. Specifically, participants felt that telehealth was not a suitable medium for physician visits in instances of new patient visits, when difficult in-depth conversations were needed, or when involving advanced clinical assessments. These instances were commonly connected to the belief held by participants that using telehealth sometimes negatively impacted the resident, family and staff experience and quality of care as well as the formation of a therapeutic relationship between residents and physicians. Care providers and care recipients alike further indicated that, in such instances, telehealth created a degree of separation among parties during consults that could not be overcome through conversation alone. The technological divide between residents, who indicated a lack of comfort with using tablets and cellular phones, and care providers created another form of separation between the parties involved in virtual physician visits. The lack of physical co-presence during telehealth appointments meant that such visits were void of face-to-face contact and personal touch, both of which were deemed important when talking about difficult topics in particular. A lack of personal touch further extended to advanced clinical assessments, where concerns were expressed regarding the impact on the quality of care. In the remainder of this section, we consider these findings in light of the existing knowledge base on telehealth use in LTC contexts and highlight some directions for future research.

Multiple recent studies have documented the challenges associated with LTC residents and physicians meeting for the first time via telehealth [27, 28], including the challenges brought on by a lack of physical touch during such initial consults [36]. The current analysis adds nuance to this existing research by integrating the perspectives of both care provider and care recipient groups. Our findings further support prior research that has highlighted that telehealth may be not appropriate for physician visits in LTC when advanced clinical assessments are needed. For example, a scoping review undertaken by Tan and colleagues (2024) identified multiple studies that documented uncertainties regarding how accurate a diagnosis could be if a nurse was describing symptoms through a telehealth medium versus if a physician were to attend in person [37]. The current study highlights the depth of this concern among LTC care staff and family caregivers in particular. Finally, other studies have echoed concerns among physicians that using telehealth for hands-on physical assessments could compromise the quality of care provided to LTC residents as physicians are unable to palpate and examine residents via phone or video [14, 29]. Participants in all groups of the current study felt that advanced clinical assessment was best done in person by physicians and should be conducted in person going forward barring any visitation restrictions that would prevent doing so.

Participants in the current study were unanimous in their belief that telehealth is not an appropriate care medium for difficult in-depth conversations about topics such as end-of-life care planning as there was a lack of personal touch and no possibility of using caring body language. There is little existing consideration of particular conversational contexts such as these related to the appropriateness of telehealth use for physician visits in LTC. As such, this is an important finding that adds critical nuance. Gaur and colleagues (2020) note the importance of ensuring adequate emotional support during advance care planning consults, citing that the social isolation typically experienced by LTC residents – which was heightened during the pandemic - may make telehealth and other virtual care mediums for such conversations more isolating rather than supportive [38]. Residents with auditory impairments will be particularly challenged in meaningfully participating in such in-depth conversations via telehealth [29], which may lead to a deeper sense of isolation and lack of emotional support among this group. Despite awareness that telehealth can decrease the ability to read non-verbal cues and provide empathetic physical touch [39], physicians’ concerns about the use of telehealth for difficult in-depth conversations captured in the current study were heavily driven by acknowledgement of the threat this care medium places on effectuating the therapeutic bond between patient and physician. The formation and enactment of a trusting, honest, and caring therapeutic relationship between patients and physicians is known to benefit patients’ quality of life [40, 41], including in the LTC context [42]. Findings from the current study raise questions about how the therapeutic potential of physician visits may be threatened in the LTC context by the use of telehealth, particularly for introductory visits and difficult in-depth conversations.

The findings from this analysis contribute to a lager utilization-focused evaluation being conducted in partnership with the Fraser Health Authority and can be used to inform future decision-making on when telehealth should and should not be used to support physician visits in the LTC context within and beyond the Fraser Health Authority region. Attentiveness to providing equitable person-centered care for residents is consistent with avoiding telehealth, when possible, for the three instances identified in this analysis. The COVID-19 pandemic saw a sweeping, rapid uptake of telehealth by Canadian physicians across multiple care sectors, some of whom have expressed concern about the lack of guidelines on when and how this care medium is best used [43]. Within LTC, there are also uncertainties about if and how to integrate specialist care into telehealth given residents’ complex care needs [19], which is something that was not addressed in the current evaluative study. Moving forward, it is essential that clear guidelines be set by physician regulatory bodies and policymakers in LTC given that Canadian physicians heavily plan to continue providing care via telehealth at a rate higher than they did prior to the pandemic [44–46]. It is anticipated that telehealth use will continue to grow specifically in LTC contexts [18], which increases the importance of having evidence-based guidelines on the appropriate use of telehealth for physician visits in LTC.

Future research exploring telehealth use in the LTC context should engage with the findings of the current analysis, and here we highlight three meaningful directions. First, our study did not explore specific indicators of care quality or health outcomes related to telehealth use in LTC for physician visits. It would be useful for future research to explore such outcomes related specifically to the three instances identified in this analysis given that care quality served as a significant driver of participants’ concern. Second, now that the roll-out of telehealth in LTC has already occurred in the Fraser Health Authority region, there may be opportunities to explore how its use can be improved to ensure that care can be provided more appropriately in a range of emergency contexts when in-person visitation is not possible or limited. For example, climate emergencies, such as wildfires and floods, are becoming increasingly common in BC and elsewhere in Canada [47]. These emergencies can impact physical access to LTC homes due to road closures or flooding, especially in rural or remote communities where crisis response resources are more limited [48]. Finally, the current analysis has focused on instances when care provider and care recipient participants deemed telehealth to be inappropriate for physician visits. It would be beneficial for future research to qualitatively explore similar topics, such as understanding if telehealth is appropriate for supporting culturally responsive and spiritual approaches to physician care. Such research would be responsive to calls to understand how to better support the culturally informed care needs of LTC residents who identify as Indigenous as current research primarily explores this topic relating to community-dwelling older adults [49].

### Strengths & limitations

This analysis has numerous strengths, two of which we wish to highlight. First, we gained perspectives from care recipients and providers alike, which provided a robust understanding of how telehealth for physician visits was experienced or perceived in the LTC context. Our use of a partner-centered approach was a second strength, where people who were closely linked to LTC supported and informed both data collection and analytical directions and enabled integrated knowledge translation. This approach was particularly useful for ensuring appropriate language use in the interview and focus group guides. With regard to limitations, we highlight three. First, a majority of residents in the Fraser Health Authority’s LTC homes had cognitive impairments that made them ineligible to participate [4]. Therefore, only the perspectives of cognitively able residents were included, which is a group that may not fully represent resident-focused experiential perspectives on telehealth use in LTC. Second, while there were options to do interviews in languages other than English, only one participant requested this. Participants in this study thus lack the full scope of ethno-cultural-linguistic diversity in the Fraser Health region. Third, only a small number of resident participants had first-hand experience with attending physician visits via telehealth. As such, the majority of their input was based on preferences for telehealth going forward instead of direct experience.

## Conclusions

Despite telehealth having various benefits for supporting physician care within LTC homes, there are limits to the appropriateness of its use. This analysis, which contributes to a larger utilization-focused evaluation study exploring the rapid roll-out of telehealth during the COVID-19 pandemic and continued telehealth use in LTC homes in BC’s Fraser Health Authority region, has identified three such limits. First, care providers and recipients agree that telehealth is not the appropriate visit medium for when residents and physicians are to meet for the first time. Second, telehealth limits the eye contact and body language that can meaningfully support difficult in-depth conversations during physician visits, such as end-of-life care planning. Third, telehealth appointments are not suitable for advanced clinical assessments if in-person options are available. Going forward, guidelines informing the appropriate use of telehealth-based physician care for providing equitable person-centered care in LTC should be created and implemented. These guidelines should integrate nuance regarding specific types of visits between residents and physicians and their appropriateness to be supported via telehealth in instances when there are also in-person options available.

## Data Availability

To maintain participants’ privacy and anonymity, interview transcripts from this study are not publicly available. Inquiries regarding gaining access to anonymized data extracts can be directed to JS.

## Abbreviations

BC: British Columbia
LTC: Long-Term Care
